# Fatty Acid Biosynthesis in Chromerids

**DOI:** 10.3390/biom10081102

**Published:** 2020-07-24

**Authors:** Aleš Tomčala, Jan Michálek, Ivana Schneedorferová, Zoltán Füssy, Ansgar Gruber, Marie Vancová, Miroslav Oborník

**Affiliations:** 1 Biology Centre CAS, Institute of Parasitology, Branišovská 31, 370 05 České Budějovice, Czech Republic; a.tomcala@centrum.cz (A.T.); jan.michalek@entu.cas.cz (J.M.); Ivana.Schneedorferova@email.cz (I.S.); zoltan.fussy@gmail.com (Z.F.); ansgar.gruber@paru.cas.cz (A.G.); vancova@paru.cas.cz (M.V.); 2 Faculty of Fisheries and Protection of Waters, CENAKVA, Institute of Aquaculture and Protection of Waters, University of South Bohemia, Husova 458/102, 370 05 České Budějovice, Czech Republic; 3 Faculty of Science, University of South Bohemia, Branišovská 31, 370 05 České Budějovice, Czech Republic

**Keywords:** *Chromera velia*, *Vitrella brassicaformis*, fatty acids, de novo biosynthesis, evolution, elongation, desaturation

## Abstract

Fatty acids are essential components of biological membranes, important for the maintenance of cellular structures, especially in organisms with complex life cycles like protozoan parasites. Apicomplexans are obligate parasites responsible for various deadly diseases of humans and livestock. We analyzed the fatty acids produced by the closest phototrophic relatives of parasitic apicomplexans, the chromerids *Chromera velia* and *Vitrella brassicaformis*, and investigated the genes coding for enzymes involved in fatty acids biosynthesis in chromerids, in comparison to their parasitic relatives. Based on evidence from genomic and metabolomic data, we propose a model of fatty acid synthesis in chromerids: the plastid-localized FAS-II pathway is responsible for the de novo synthesis of fatty acids reaching the maximum length of 18 carbon units. Short saturated fatty acids (C14:0–C18:0) originate from the plastid are then elongated and desaturated in the cytosol and the endoplasmic reticulum. We identified giant FAS I-like multi-modular enzymes in both chromerids, which seem to be involved in polyketide synthesis and fatty acid elongation. This full-scale description of the biosynthesis of fatty acids and their derivatives provides important insights into the reductive evolutionary transition of a phototropic algal ancestor to obligate parasites.

## 1. Introduction

**Figure 1:**
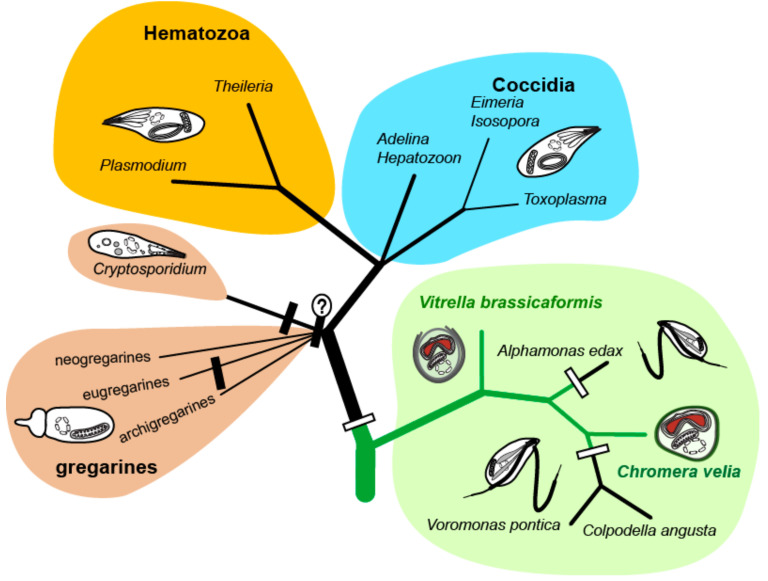
Phylogenetic tree of chromerid algae and apicomplexan parasites. The white rectangles indicate the loss of photosynthesis, the black ones indicate the loss of plastid.

The phototrophic alveolates *Chromera velia* [1] and *Vitrella brassicaformis* [2] (referred to as chromerids) were isolated from stony corals in Australia using methods for the isolation of intracellular symbionts. Both chromerids can live as free-living algae without hosts in laboratory cultures; however, they are associated with corals in the wild [1,2,3,4]. Although they were found in the larvae of several coral species [5], they have never been detected in adult corals [6]. It was speculated that chromerids live as mutualists like symbiotic dinoflagellates of the genus *Symbiodinium* [1,7]. However, the latest transcriptomic survey of coral larvae experimentally infected by *C. velia* opens the possibility that the alga can be their facultative or even accidental parasite [8]. The environmental detection of uncultured and undescribed chromerids and related organisms (ARL-V) also implies their probable commensal or parasitic relationship to corals [9]. Such finding is congruent with the accumulating evidence supporting the common ancestry of phototrophic chromerids and parasitic apicomplexans [3,4,10,11,12,13,14]. Analyses of plastid [11] and mitochondrial [15] and nuclear genomes [10,13], together with the determination of ultrastructural characters such as cortical alveoli, four-membrane plastid envelopes [1], the presence of a pseudoconoid in *C. velia* [16], and zoosporogenesis by budding in *V. brassicaformis* [17], support the unique phylogenetic position of chromerids at the root of the parasitic Apicomplexa. Further molecular phylogenetic and phylogenomic analyses have placed the predatory colpodellids within the polyphyletic chromerids (Figure 1), constituting a group named Apicomonada [18] or chrompodellids [10].

Fatty acids (FAs), carboxylic acids with aliphatic chains, represent a fundamental component of biological lipids and can be divided into several categories according to their structure [19]. Here, we focus on the most abundant subclass—straight-chained saturated and unsaturated FAs with a terminal carboxylic acid group. Generally, FAs are synthesized by repeated addition of two-carbon units to a growing carboxylic acid chain attached to the acyl carrier protein (ACP) [20]. In cellular organisms, the enzymes responsible for the de novo synthesis of FAs are either the type I FA synthases (FAS-I) typically found in heterotrophic eukaryotes or the type II FA synthases (FAS-II) usually referred to as bacterial-type FAS-II. Both pathways contain identical reaction steps but they substantially differ in the architecture of the responsible enzymes. In eukaryotes, cytosolic FAS-I is a huge multi-modular enzyme [21], while FAS-II consists of individual separate enzymes located in the plastid [22,23,24]. Modules in the FAS-I multi-modular enzymes have the full set of domains needed for the attachment of FA carbon units. If such a multi-modular enzyme contains an incomplete module lacking some of the domains (often acyltransferase) or has some additional domains (e.g., methyltransferase), it usually has a different function. Such giant multi-modular enzymes known as polyketide synthases (PKSs) are responsible for the synthesis of antimicrobial compounds called polyketides (PK) [25]. It was proposed that FAS-I derived from FAS-II via a gene fusion [25]. Plants and algae primarily use bacterial FAS-II located in the plastid, with the involved enzymes encoded in the nuclear genome [24,26]. Many algae also possess FAS-I/PKS-like enzymatic equipment. Their function is still enigmatic; however, they likely do not act as FA synthases [25]. Various lineages of apicomplexan parasites differ in the presence of the particular FAS system. Most apicomplexan parasites possess the apicoplast-located FAS-II for de novo production of FAs [20,27,28], with Haemosporidia such as *Plasmodium* utilizing only this prokaryotic type of the synthesis. The coccidians *Toxoplasma gondii* or *Eimeria tenella* were described to use the apicoplast FAS-II system and the cytosolic FAS-I in parallel, with life stage-specific FA metabolism [20,29,30,31]. However, the apicomplexan minimalists *Theileria parva* and *Babesia bigemina* completely lost all the enzymes for FA synthesis and scavenge all FAs from their hosts [20]. Current findings suggest independent multiple FAS-II losses in apicomplexans and their relatives [32].

Last but not least, intestinal parasites of the genus *Cryptosporidium* do not possess the apicoplast at all and lack any de novo FA synthesis. The huge FAS-I-like multi-modular enzyme coded in the *Cryptosporidium parvum* genome is responsible for the elongation of palmitic acid acquired from the host [13,20,33]. The high divergence of the apicomplexan sources of fatty acids reflects different stages of reductive evolution driven by adaptations to diverse hosts [13]. Genomic analysis performed by John and co-workers [34] revealed that some representatives among apicomplexans such as *C. parvum*, *E. tenella*, and *T. gondii* possess PKS genes. However, the presence of PKS genes is not universal among apicomplexans. For instance, in the genomes of *Plasmodium falciparum* and *T. parva*, these sequences are lacking [34].

Here, we studied FAs and their synthesis in the chromerids *C. velia* and *V. brassicaformis* by the combination of analytical chemistry and genomics methods. We compared chromerid and apicomplexan FA biosynthesis and propose a scenario of their evolution.

## 2. Materials and Methods

### 2.1. Growth Experiments

Cultures of *C. velia* and *V. brassicaformis* were grown in f/2 medium in 25 cm^2^ flasks under artificial light, with a photoperiod of 12 h light/12 h dark and light intensities of 30–50 μmol m^−2^ s^−1^ at a temperature of 26 °C. As an inoculum, 1 mL of *C. velia* (1 × 10^6^ cells/mL) or 5 mL of *V. brassicaformis* (0.3 × 10^6^ cells/mL) stationary culture was added to each flask with 40 mL of f/2 medium. The cultures were grown for 50 days and harvested via centrifugation. Pellets for lipid extraction were stored at −20 °C.

The cultures of *C. velia* were treated with Triclosan (TCI Europe N. V., Zwijndrecht, Belgium) [35] in the same cultivations conditions as mentioned above. Triclosan was diluted in dimethyl sulfoxide (DMSO, Sigma Aldrich, St. Louis, Missouri, USA). The inhibitor was added to cultures after 14 days, at six concentrations in the media (0.02, 0.04, 0.08, 0.133, 0.333, and 0.667 µM). The densities of the cultures were periodically measured by a spectrophotometer (Infinite^®^ 200 PRO, Tecan, Männedorf, Switzerland) over the course of 36 days. Fifty-day-old cultures were harvested by centrifugation in 50 mL flacons (Hettich Micro 22R centrifuge with rotor radium 100 mm, set to 6000 rpm at the of temperature 4 °C). The obtained pellets were split; the first part was stored at −20 °C for fatty acid analyses; the second part was subjected to transmission electron microscopy (TEM).

To investigate the effects of various levels of inorganic nitrogen in the media, *C. velia* cultures were grown in standard conditions, as mentioned above (light, temperature, cultivation flasks). Flasks containing 120 mL of f/2 solution with four different concentrations of NaNO_3_ (from 0.000 to 0.062 g of pure nitrogen per 1 L of f/2 medium) were inoculated using 1 mL of stationary-stage culture of *C. velia* (1 × 10^6^ cells/mL). The highest concentration of nitrate in this experiment was five times higher than in the regular f/2 medium. The cultures were harvested by centrifugation after 30 days. The dry pellets were weighed and investigated using gas chromatography coupled with a flame ionization detector (GC/FID).

### 2.2. Genomic Search

BLAST search and InterProScan in the publicly available dataset of predicted proteins [13] (CryptoDB; https://cryptodb.org/cryptodb/) were used to search for the enzymes of the FA biosynthesis in *C. velia* and *V. brassicaformis*. The obtained sequences were complemented with homologs found in other public databases (NCBI, KEGG, JGI) and aligned using MAFFT [36]. The alignment was manually edited by excluding gaps and ambiguously aligned positions using Geneious [37]. Phylogenetic trees were computed using the maximum likelihood method (RAxML; [38] and Bayesian inference (MrBayes 3.2.4.; [39,40]. The subcellular locations of the enzymes were predicted using TargetP [41,42], SignalP [43], and ASAFind [44]. Localizations were established with the support of multiple predictors together; previously published data were also considered. For phylogenetic analyses, we took advantage of computational resources freely provided by the CIPRES Science Gateway [45].

### 2.3. Fatty Acids—Gas Chromatography Coupled with a Flame Ionization Detector (GC/FID)

Lipids were extracted following the chloroform–methanol solution method [46] modified by Košťál and Šimek [47], with additions described by Tomčala et al. [48]. Briefly, homogenized algal samples were dried, weighed, and extracted. The raw extracts in chloroform were dried and resolved in 500 µL of chloroform. Then, 100 µL aliquots were transformed into methyl esters of fatty acids (FAMEs) to allow for GC analyses. For this purpose, sodium methoxide was employed as a transesterification reagent, as described previously [49]. FAMEs were then analyzed by GC/FID using a gas chromatograph GC-2014 (Shimadzu, Shimadzu Scientific Instruments Inc., Kyoto, Japan) equipped with the column BPX70 (SGE, SciTech, Prague, Czech Republic) and an autosampler with the injector AOC—20i (Shimadzu), with the settings according to a previous work published by Tomčala et al. [48]. For the identification of FAs, a mixture of 37 standards purchased from Supelco Inc. (Bellefonte, Pennsylvania, USA) was used.

### 2.4. Transmission Electron Microscopy

Pellets were transferred to HPF (High Pressure Freeze) carriers (Leica Microsyst. Vienna, Austria), then f/2 medium with 20% (*w/v*) bovine serum albumin (BSA) was added to fill the carriers completely. The samples were immediately frozen using the high-pressure freezer Leica EM PACT2. The frozen samples were incubated with 2% osmium diluted in 100% acetone in a freeze substitution unit, with the following substitution program: −90 °C for 96 h; then the temperature was increased to −20 °C (with a slope of 5 °C/h); after 24 h, the temperature was raised (3 °C/h) to the final temperature of 4 °C. After 18 h of incubation, the samples were washed with acetone and infiltrated by 25%, 50%, 75% EMBed 812 resin (EMS)/acetone solutions, 1 h at each step, at room temperature. Finally, fresh resin was added, and the specimens were placed overnight in a vacuum desiccator. The resin was allowed to polymerize at 60 °C for 48 h. Images were obtained by a transmission electron microscope (JEM 1010, JEOL Inc., USA) with an acceleration voltage of 100 kV [16].

## 3. Results

### 3.1. De novo Fatty Acid Synthesis

**Figure 2:**
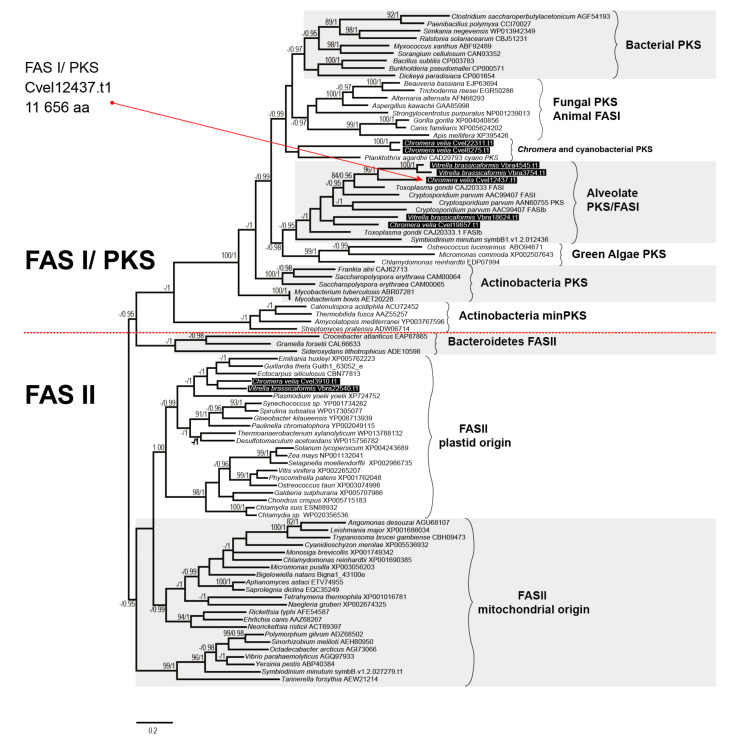
The phylogenetic tree as inferred from ketoacyl synthase domains to distinguish type I and type II fatty acid synthases. Bootstrap for 1000 repetitions and Bayesian pp values (BS1000/1M, CAT/LG models) are displayed above or below the branches. FAS, fatty acid synthase, PKS, polyketide synthase.

**Figure 3:**
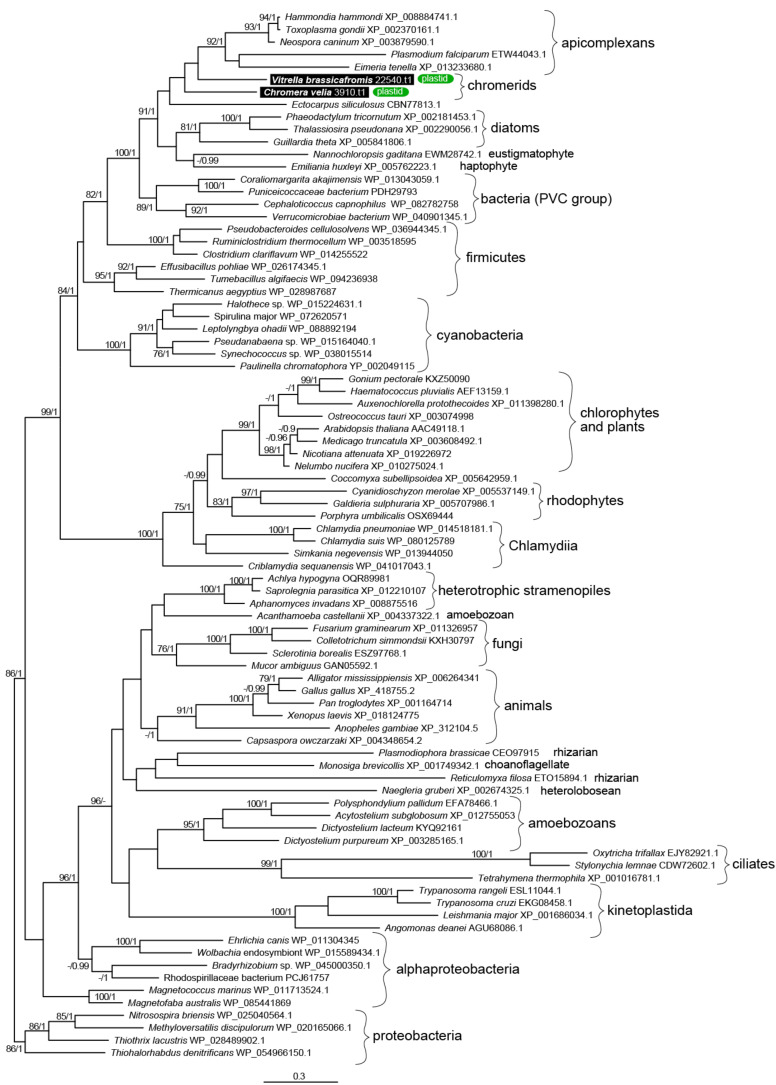
The detailed maximum likelihood phylogeny of ketoacyl synthase domains of type II fatty acid synthases. Bootstrap for 1000 repetitions and Bayesian pp values (BS1000/1M, CAT/LG models) are displayed above the branches. Predicted subcellular locations are shown.

**Figure 4:**
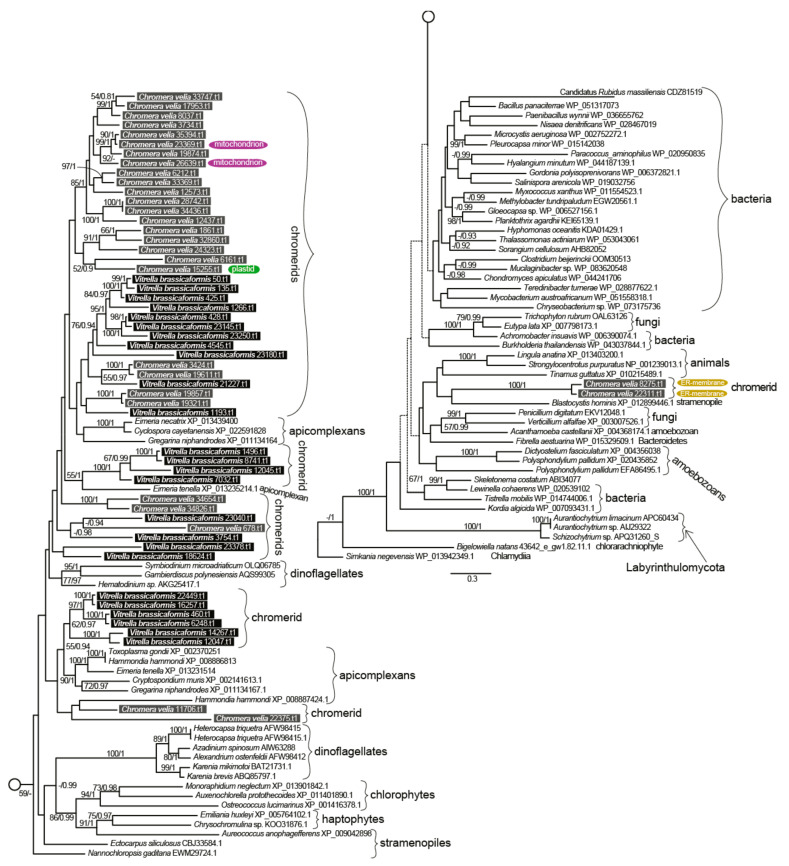
The detailed phylogeny of ketoacyl synthase domains of type I fatty acid synthases and polyketide synthases. Bootstrap for 1000 repetitions and Bayesian pp values (BS1000/1M, CAT/LG models) are displayed above the branches. Predicted subcellular locations other than cytosolic are shown.

The presence of a FAS-II in *C. velia* was first described by Dahmen et al. [50] and Woo et al. [13], who indicated the presence of FAS-I and FAS-II in both chromerids. We searched in the chromerid genomic and transcriptomic data (CryptoDB) for all the enzymes possibly involved in the synthesis of FAs and their derivatives (Table 1, Table 2, Table 3, Table 4 and Table 5). In addition to the canonical FAS-I and FAS-II enzymes, the genome contains a large number of genes likely encoding ketoacyl synthases (36 genes), short-chain dehydrogenases (73), or acyltransferase domains (29), present in various gene arrangements, often in a form of multi-modular enzymes with unknown putative function in FA or PK synthesis (Table 1, Table 2, Table 3, Table 4 and Table 5). We performed a phylogenetic analysis based on ketoacyl synthase (KS) domains to distinguish FAS-I and FAS-II enzymes of chromerids and further calculated detailed phylogenies of FAS-I- and FAS-II-type enzymes (Figure 2, Figure 3 and Figure 4). Both chromerids possess numerous giant multi-modular enzymes in sizes ranging from 1000 to 11,000 amino acids composed of 1–5 modules and classified as putative FAS-I/PKS-I [13]. In general, KSs of type II FAS show two main lineages, i.e., α-proteobacterial and cyanobacterial, with the latter genes involved in plastid-located KSs in chromerids (Figure 3). Ketoacyl synthases from FAS-I/PKS-I enzymes of chromerids, apicomplexans, and dinoflagellates form a sister group to PKSs responsible for the production of polyketide-based algal toxins found in dinoflagellates, chlorophytes, haptophytes, and stramenopiles (Figure 4). Since the evolutionary conversion between FAS and PKS function can be caused by a single amino acid substitution affecting the functionality of the participating domain [51], it is hard to identify them just by a phylogenetic signal. One distant clade of type I multi-modular FAS-like enzymes is formed by two genes of *C. velia* (Cvel_8275.t1, Cvel_22311.t1). These two genes share a multiple-domain architecture reminding a single module of PKS as well but they are interestingly fused with an N-terminal structure related to the AcrB-like domain family (multidrug efflux pump) or the sterol-sensing domain, which, together with a predicted N-terminal signal anchor (Table 1, Table 2, Table 3, Table 4 and Table 5) implies a probable association of the enzyme with the endoplasmic reticulum (ER).

### 3.2. Elongation

**Figure 5:**
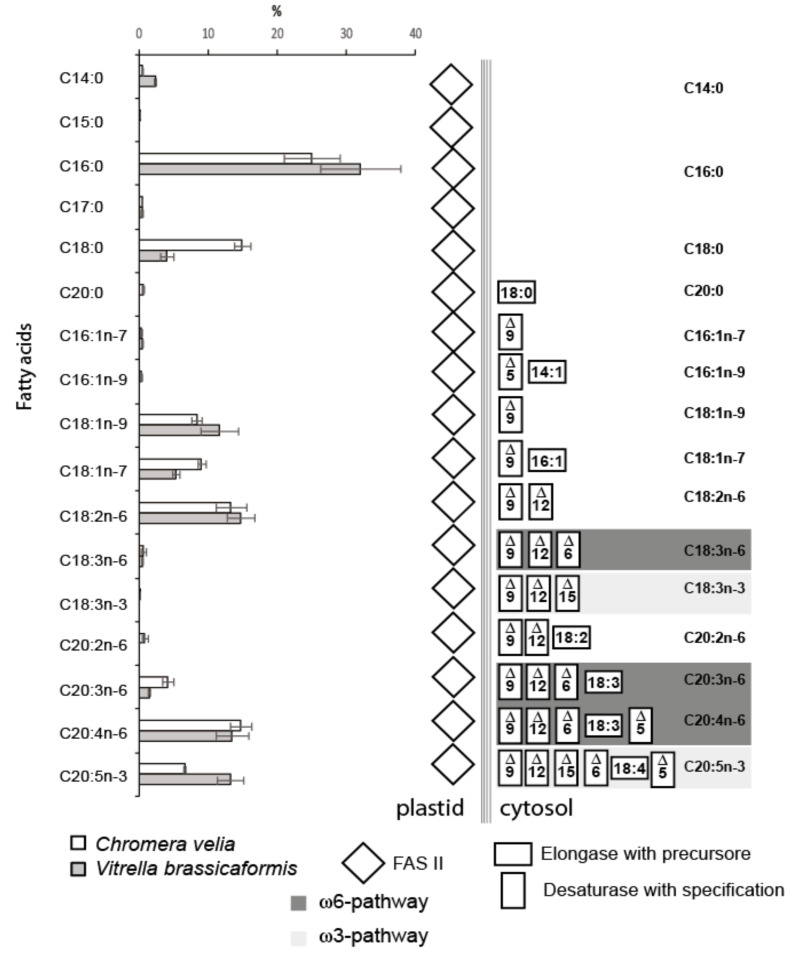
The fatty acid composition of both algae obtained by GC/FID (*Chromera velia* 350 ± 38 mg/g and *Vitrella brassicaformis* 4 ± 0.8 mg/g dry mass) and enzymatic repertoire for particular fatty acid synthesis and modification (*n* = 5). The concentrations of FAs are displayed in percentages with standard deviations. *C. velia* tends to accumulate storage lipids and contains approximately 100-fold higher FAs concentrations than *V. brassicaformis*. The absolute concentrations of individual fatty acids can be calculated from absolute FAs concentrations values.

**Figure 6:**
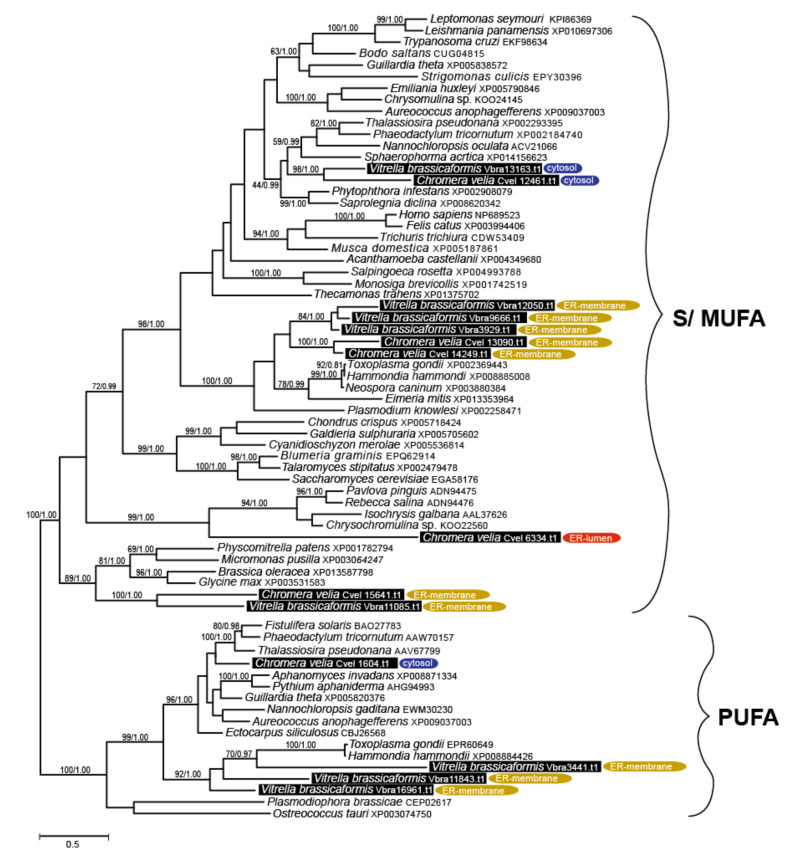
Bayesian phylogenetic tree inferred from elongase domains (70 taxa, 188 amino acid residues). Numbers above the branches display ML bootstrap/Bayesian PP support (BS1000/3M, CAT/LG). Predicted subcellular locations are displayed.

The repertoire of FA elongases (Table 1, Table 2, Table 3, Table 4 and Table 5) differs between the two chromerids, even though their detected FA spectra are highly similar (Figure 5). The class-specific sequence motifs [52] and phylogenetic signal in desaturase domains allowed us to recognize two major functional classes (Figure 6):

(i) Elongases specific for the saturated and monounsaturated fatty acids (S/MUFAs), forming four different orthologous groups. The total number of genes in each species is four, but their phylogenetic distribution within the tree and their predicted intracellular locations appeared to be species-specific. The first group predicted as cytosolic in location and counting one gene per species resulted to branch with stramenopiles. The second distant clade, putatively localized to the ER membrane, was found to be composed of *V. brassicaformis* (three genes) and *C. velia* (two genes). The remaining two S/MUFA elongases of *C. velia* and *V. brassicaformis* share origin with chlorophytes and plants at the very base of the S/MUFA group. A single distinct elongase of *C. velia* was found to form a sister long branch to haptophytes.

(ii) Elongases specific for poly-unsaturated FAs (PUFA). A single gene coding for a cytosolic elongase (Cvel_1604.t1) of PUFA was found in *C. velia*, whereas *V. brassicaformis* appeared to encode three paralogous enzymes putatively located in the ER membrane (Table 1, Table 2, Table 3, Table 4 and Table 5). The single cytosolic PUFA desaturase of *C. velia* clusters with stramenopiles, while the three ER-located elongases from *V. brassicaformis* branch together with apicomplexans (Figure 6).

### 3.3. Desaturation

**Figure 7:**
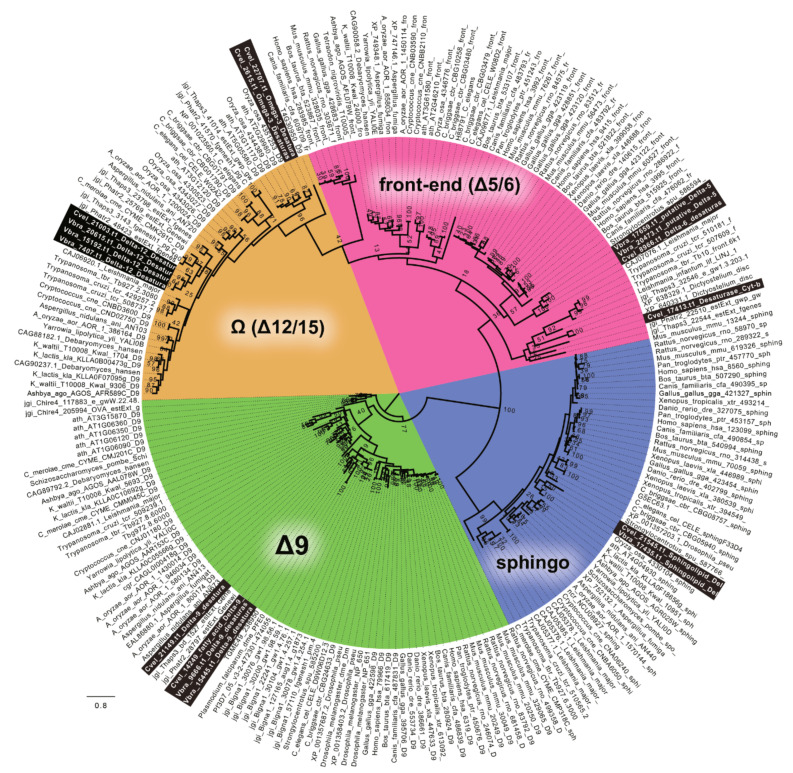
The RaxML tree resolving major functional classes of FA desaturases within various genomes, as described by Gostincar 2010 [50]. The chromerid genomes contain all types of desaturases. Detailed phylogenies of particular classes are displayed below.

The desaturation of FAs is accomplished by desaturases of four different major classes defined by the specific products of their action. In both chromerid genomes, we identified desaturases of all functional classes. Most of the genes appeared to have no detectable N-terminal-targeting pre-sequence (Table 1, Table 2, Table 3, Table 4 and Table 5), with omega desaturases making an exception (Table 1, Table 2, Table 3, Table 4 and Table 5). Therefore, we presume that most desaturation processes likely take place in the cytosol. To distinguish the main functional enzyme groups, we constructed a maximum likelihood phylogeny (Figure 7) based on the dataset of desaturase domains from various organisms previously published by Gostincar et al. [53]. Using this initial phylogeny-based sorting, we built more detailed phylogenies focused on particular desaturase classes (Figures S1–S3).

Sphingolipid desaturases were not an object of this study. However, they were included in the phylogeny (Figure 7) to test the completeness of the dataset. Regarding the evolution of desaturases, Δ-9 desaturases are generally considered as the ancestral group. The Δ-9 class is represented by two genes in both *C. velia* and *V. brassicaformis*. The likelihood tree (Figure S1) suggested their origin within the SAR clade (Stramenopila + Alveolata + Rhizaria). The highly divergent gene Cvel_21149.t1 forms the long branch far distant from the SAR group or any sequence data deposited in databases. The prediction suggests a highly possible plastid targeting of Cvel_21149.t1 (Table 1, Table 2, Table 3, Table 4 and Table 5).

**Figure 8:**
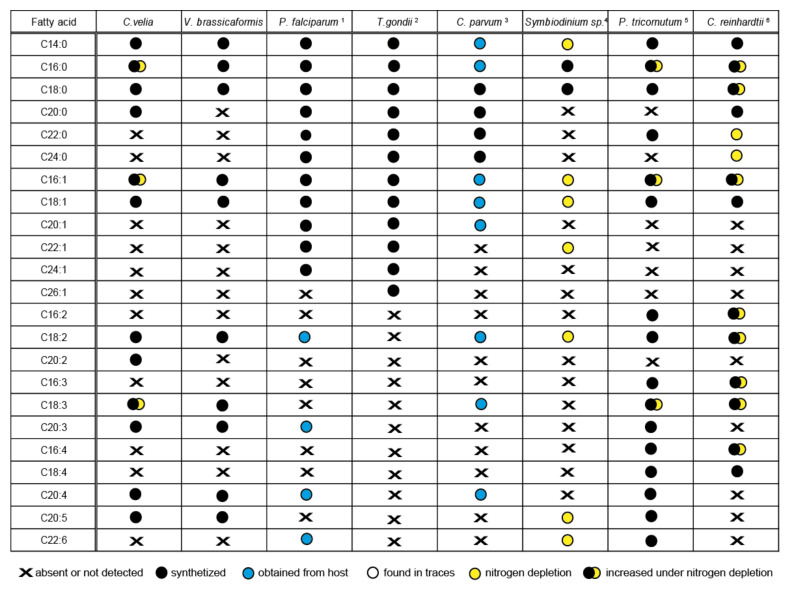
Comparison of the fatty acid profile of particular organisms and the origin of particular fatty acids. Liver-stage of *Plasmodium*, intracellular stages of *Toxoplasma gondii* 1) Botté et al., 2013 [54], 2) Ramakrishnan et al., 2012, 2015 [52,55], 3) Mitschler et al., 1994 [56], 4) Weng at al., 2014 [57], 5) Siron et al., 1989; Popko et al., 2016 [58,59], 6) James et al., 2011; Puzanskiy et al., 2015 [60,61]. The position of the last double bond was not specified in some mentioned publications; therefore, it is not included in the table.

The Δ-5/6 (front-end) enzyme class was found to be represented by two enzymes in each chromerid genome. The phylogenetic position of Cvel_17413.t1 is not fully resolved, likely due to high sequence divergence. The remaining genes form *C. velia* (1) and *V. brassicaformis* (2) coding for Δ-5/6 (front-end) desaturases constitute a monophyletic sister group to their chlorophyte counterparts, branching in the proximity of perkinsids, rhizarian, haptophyte, kinetoplastids, stramenopiles, and choanoflagellate in the well-supported eukaryotic clade. TargetP predicted Vbra_20473.t1 as mitochondrially located (Figure 8, Figure S2).

The Δ12-desaturase group found in *C. velia* forms two distinct clades: the first clade consists of two genes from *C. velia*, branching with plants and cyanobacteria, suggesting its plastid origin. The second clade is composed of one desaturase from *C. velia* together with three genes from *V. brassicaformis* and branches between stramenopiles and ciliates, within a major clade composed mainly of SAR group members, haptophytes, and cryptophytes (Figure S3). The N-terminal pre-sequence detected in the desaturase Cvel_22707.t1 sharing its origin with plants displays a bi-partite architecture characteristic for targeting complex plastids (Table 1, Table 2, Table 3, Table 4 and Table 5). The desaturase Vbra_7407.t1 shows only a hint of mitochondrial transit peptide, with low probability and reliability for the prediction. Thus, we considered this structure to be an artifact and this whole clade of chromerid desaturases as cytosolic.

### 3.4. Fatty Acid Profile

The list of all FAs detected in chromerids and their abundances are shown in Figure 5. The outcome of the described complex enzymatic machinery is a broad spectrum of FAs that are variable in abundance, number of carbons, number and position of double bonds. *C. velia* and *V. brassicaformis* have a high content of palmitic acid (C16:0), covering 25% and 33% of all FAs in the two algae. The FA profiles of chromerids are very similar to each other, with two exceptions: the occurrence of stearic acid (C18:0) in *C. velia* is three times higher than in *V. brassicaformis*, and *V. brassicaformis* contains two times more eicosapentaenoic acid (C20:5n-3) (EPA) than *C. velia*. The detected FAs (Figure 8, Figure 5) indicate the presence of both PUFA pathways ω6 (animals) and ω3 (algae), resulting in a chain of 20 carbon units with five double bonds (EPA). However, the last enzyme of the ω6 pathway, Δ-17desaturase, is missing in both algae in correlation with their high content of arachidonic acid (C20:4n-6). It seems that *V. brassicaformis* engages the ω3 pathway preferentially, unlike *C. velia*, which employs both pathways at a similar level (Figure 5, Figure S4a,b). The precursors of both pathways occur at very low levels or below the limit of detection (e.g., C18:3n-6, C18:3n-3, and C18:4n-3). The presence of C20:2n-6 among FAs of *C. velia* indicates an alternative ω6 pathway (Figure 3, Figure S4a). In contrast, C20:2n-6 is not present in *V. brassicaformis*, with no obvious alternative ω6 pathway (Figure S4b). The high variability of FAs detected in chromerids is also highlighted by the presence of oleic FA (C18:1n-9) and cis-vaccenic acid (C18:1n-7). These two FAs are based on palmitic acid, modified by only two enzymes, Δ-9 desaturase and elongase. C18:1n-9 is synthesized by elongation of palmitic acid (C16:0) to stearic acid (C18:0) and subsequent desaturation at the 9th carbon position, in contrast to the synthesis of cis-vaccenic acid, which begins with the desaturation of palmitic acid at the 9th carbon position followed by elongation, resulting in a shift of the double bond to the 7th position (for details, see Figure S4a,b).

After 20 days of triclosan treatment, all cultures treated with a concentration higher than 40 µM stopped growing (Figure S5), bleached, and died. Cells treated with solvent control (DMSO) were not affected (data not shown). At the concentration of 40 µM, triclosan drastically inhibited the production of unsaturated FAs and slightly affected the abundance of saturated FAs. When the inhibitor concentration was increased up to 330 µM, we observed the vanishing of unsaturated FAs and a dramatic downregulation of C16:0. No such drastic decrease was observed in the production of C18:0 (Figure S6). Even a slightly higher concentration of triclosan in the medium caused a fatal collapse of the whole membrane system, as seen by transmission electron microscopy (Figure S7a,b).

Our experiment with nitrogen repletion and depletion showed changes in the lipid profiles of *C. velia*: FAs up to C18 increased in abundance by approximately 14% during nitrogen depletion, while FAs longer than C18 were more abundant during nitrogen repletion (Figure S8). The modulation of the FA profile by environmental conditions was also described [62].

## 4. Discussion

**Figure 9:**
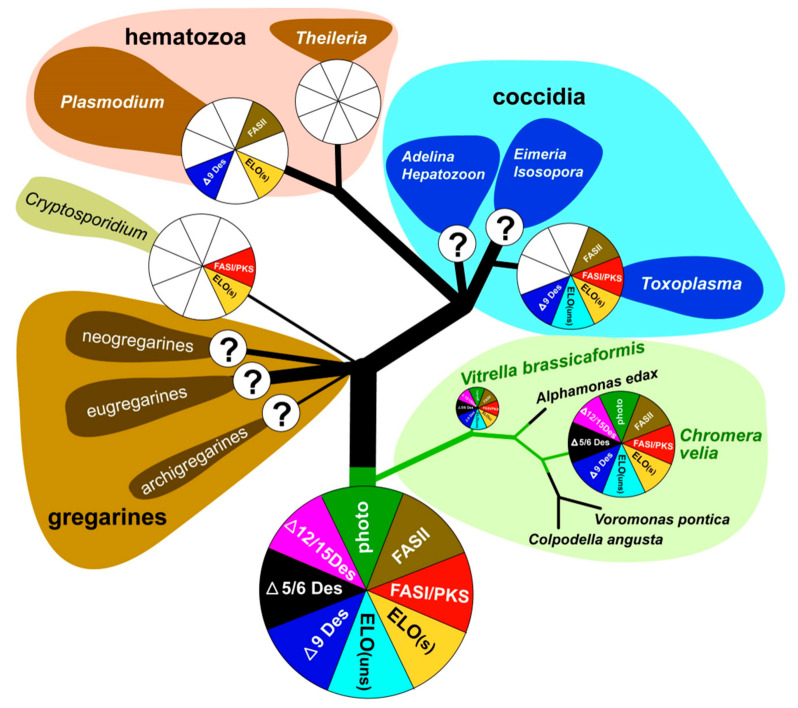
Overview of FAS evolution in chromerids and apicomplexan parasites.

Both types of FAS have been detected in chromerids, *T. gondii*, and other coccidians (Figure 9). Type I FAS enzymes are preserved mainly in organisms that have lost their plastids, such as *Cryptosporidium*, or in those that never had plastids. It is possible that in plastid-bearing organisms, the rarely present cytosolic FAS-I is employed in the synthesis of some special FAs; however, it is more likely that FAS-I multi-modular enzymes in chromerids and coccidia are involved in the elongation of FAs rather than in de novo synthesis, as suggested for FAS-I enzyme in *C. parvum*. It was nicely shown that the multi-modular FAS-I enzyme from this plastid-lacking intestinal apicomplexan parasite is responsible for the elongation of short fatty acids scavenged from the host [33]. Since the acetyl-CoA (coenzyme A) carboxylase (ACC) from *C. parvum*, which is used for elongation and not FA de novo synthesis branches together with the cytosolic ACCs from *T. gondii* and chromerids (Figure S9), they can hypothetically do the same job. Although chromerids, coccidians, and *Cryptosporidium* are known to encode numerous FAS-I-like multi-modular enzymes in their genomes, only some of them are believed to be involved in FAS metabolism. Others more likely represent evolutionary related genes coding for type I polyketide synthases (PKS-I) responsible for the synthesis of polyketides, secondary metabolites known to act as antimicrobial compounds and toxins, such as cyanobacterial toxins, dinoflagellate red-tide toxins (e.g., brevetoxin), mycotoxins (e.g., aflatoxin), and even some compounds used as antibiotics (geldanamycin, doxycycline, and others) [51]. All these enzymes contain at least one incomplete module lacking an acyltransferase (AT) domain, with the absence of this domain being considered as a hallmark of most algal type I PKSs [25]. Furthermore, one and two multi-modular FAS-like enzymes in *C. velia* and *V. brassicaformis*, respectively, contain additional methyltransferase (MT) domains, which is also typical for PKSs [63].

The coccidian *T. gondii* contains at least one FAS-I enzyme with four complete modules (9940 amino acids) [57] and PKS-I enzymes [22]. *C. parvum* contains, in addition to the FAS-I multi-modular gene with three modules, also a PKS gene, which consists of seven modules containing 13,411 amino acids. The exclusive feature of all FAS-I and PKS-I in apicomplexans is likely the formation of highly resilient oocysts (*Cryptosporidium*, *Eimeria*, *Toxoplasma*) [30]. Since these enzymes have affinity to similar substrates, it is speculated that FAS-I, together with multi-domain PKSs, collaborate to produce special lipids constituting the oocyst membrane. This cooperation between FAS-I and PKS-I has also been reported in certain dinoflagellates, fungi, and bacteria [22]. A durable cell wall is also characteristic of certain life stages of *C. velia* and *V. brassicaformis* [1,2]. Although most multi-domain FAS-I/PKS-I enzymes in chromerids show sequence structures typical for PKS rather than FAS-I, both algae have two different ACCases (Figures S9 and S10). The first one is related to the plastid originated and located ACCases, similar to those of other apicomplexans, dinoflagellates, cryptophytes, haptophytes, and chlorophytes. The second one, which is likely cytosolic, branches with that of *T. gondii* and *C. parvum*. Since it was proposed that cytosolic acetyl CoA carboxylase could be a source of malonyl-CoA for the elongation of fatty acids [22,64], FAS-I in apicomplexans may be involved in elongation. As canonical elongases are also present in chromerid genomes, FAS-I multi-modular enzymes can be involved in FA elongation in specific life stages, like in *T. gondii* [65] and *C. parvum* [33].

As mentioned previously [13,58], chromerids are equipped with cytosolic FAS-I multi-modular enzymes originating from the eukaryotic heterotrophic host (exosymbiont) and with plastid-located FAS-II, which was acquired during the complex endosymbiotic event from the endosymbiont (Table 1, Table 2, Table 3, Table 4 and Table 5). This pathway arrangement correlates with the elevated expression of plastidial ACCase during nitrogen depletion, which has been described in *C. velia*, and in the haptophyte *Isochrysis galbana* [64]. The tendency of chromerids to switch metabolism towards storing energy in the form of lipids, which is enhanced in nitrogen-poor media, is shared with other algae like the symbiotic dinoflagellate *Symbiodinium* spp. [59], the eustigmatophyte *Nannochloropsis gaditana* [66], the diatom *Phaeodactyllum tricornutum* [60,61,67], the rhodophyte *Cyanidioschyzon merolae*, and the chlorophytes *Chlorella vulgaris* [68] and *Chlamydomonas reinhardtii* [55,69,70]. The complete FAS-II apicoplast-located system was recorded in *T. gondii*, *P. falciparum*, and *E. tenella* [22,29,54,71,72,73,74,75], and all these apicomplexans use it for the de novo synthesis of saturated FAs [20,29,57,76]. The apicomplexan minimalist *Cryptosporidium* does not possess the apicoplast, consequently lacks the FAS-II pathway, and fully depends on FAs scavenged from the host and in-house elongation [33]. The blood parasites *Babesia* and *Theileria* completely lack FAS and modification enzymes, despite the presence of the apicoplast in their cells [20,22,33] (Figure 9). Importantly, the FAS-II pathway was found in all organisms bearing red algal-derived plastids [23]. Based on experiments with the reportedly selective inhibitor of FAS-II triclosan [77], it was initially suggested that it is the FAS-I system which is responsible for FA de novo synthesis in *C. velia*, while FAS-II is involved in elongation and desaturation of primary FAs to final PUFAs arising from both ω-3 and ω-6 pathways [13]. A similar FA biosynthetic and modulatory system was initially proposed for *T. gondii*, again partly based on experiments with triclosan and other selective inhibitors. Mazumdar and Striepen [30] deduced that *T. gondii* employs FAS-I in the de novo synthesis of FAs and FAS-II in lipoic acid synthesis. However, experiments performed in the absence of selective inhibitors finally suggested that the de novo synthesis of FAs in *T. gondii* is maintained exclusively by the apicoplast-located type II FAS pathway [54,57]. After several of our experiments with triclosan and considering the above-mentioned published results, we doubt its inhibitory specificity. The target of triclosan is supposed to be the enoyl reductase domain, which plays an indispensable role in both FAS-I and FAS-II systems. Thus, the inhibition should affect both types of synthesis [78]. Since the enzymatic steps involved in the elongation process are like those of FAS-I and II, but the growing chain is held by CoA instead of ACP [30], we hypothesize that lower concentrations of triclosan mainly affect cytosolic enoyl reductases, while the plastidial ones are likely protected by multiple plastid membranes. It seems that plastid membrane protection is not efficient at triclosan concentrations of 80 µM and higher. A fatal collapse of the whole membrane system, which is apparent in the TEM pictures (Figure S6), could be induced by the inhibition of plastidial enoyl reductase. We assume that the specificity of triclosan is at least questionable and that this compound non-specifically inhibits elongases and desaturases in the cytosol and endoplasmic reticulum rather than FA synthesis in the plastid. Therefore, we propose that the de novo synthesis of FAs occurs in the plastid using a prokaryotic type of fatty acid synthesis, like in other phototrophic eukaryotes. The FAS-II pathway was found responsible for FA synthesis in all organisms bearing red algal-derived plastids [23]. It also appears reasonable that one of the most energetically expensive processes, for which also reduction equivalents are needed, is associated with the plastid, the main ‘power station’ and producer of reduction equivalents of the phototrophic cell. Taking all the above-mentioned facts into account, we propose that chromerids synthesize at least the major pool of fatty acids in the plastid using the FAS-II system.

Sequence identification of the enzymes involved in FAS and their localization results (Table 1, Table 2, Table 3, Table 4 and Table 5) together with GC/FID analysis of FAs (Figure 5) provided enough information for proposing a model for FA biosynthesis, elongation, and desaturation in chromerids (Figure S4a,b). The primary sources of energy and carbons for the synthesis of FAs in chromerids are photosynthesis and the Calvin cycle, as described in plants [56,79]. Based on our findings, we suggest that the plastid-located FAS-II system de novo synthesizes short saturated FAs—mainly C16:0, followed by C18:0 and C14:0 acids—and exports them to the cytosol in the form of FAs or associated with ACP. FA–ACP complexes are then modified by the set of desaturases and elongases. Most enzymes participating in the final steps of FA synthesis are either located in the cytosol or associated with the membrane of the endoplasmic reticulum, with the exception of one plastid-targeted ∆9 desaturase (Cvel_21149), one plastid-targeted ω-desaturase (Cvel_22707), and one front-end desaturase (Vbra 20473), likely located in the mitochondrion (Table 1, Table 2, Table 3, Table 4 and Table 5). Plastid-targeted ∆9 and ω-desaturases were also described in plants [24] and are the only desaturases retained in *Plasmodium* lacking any traces of FAS-I system (Figure 9). It should also be pointed out that chromerids do not produce C16 fatty acids with more than one double bond. Such poly-unsaturated C16 fatty acids are common in a broad spectrum of non-chromerid algae; for example, the diatom *P. tricornutum* and the green alga *C. reinhardtii* contain these FAs even with four double bonds in the 16 carbons chain (Figure 8). The presence of an alternative pathway to produce EPA in *C. velia* is very common and has been previously reported in the chlorophyte *Pariechloris incista* [80]. Furthermore, the diatom *P. tricornutum* has three [81], and *Euglena gracilis* has four different pathways to produce EPA [82]. The absence of any alternative EPA synthesis pathway in *V. brassicaformis* is even more interesting (Figure S4b). Chromerids lack long FAs (longer than C20), like other mentioned eukaryotic phototrophs, except for *P. tricornutum*, containing polyunsaturated FAs (22:6) (Figure 8).

Chromerids encode a broad spectrum of enzymes involved in the synthesis of short-chain saturated FAs and their derivatives. The evolutionary diagram in Figure 9 displays differences between chromerids and apicomplexans, which have completely lost Δ5/6 and Δ12/15 desaturases essential for the biosynthesis of PUFAs. However, previous works dealing with fatty acid determination showed the occurrence of PUFA in *P. falciparum* [76] and *C. parvum* [83] (for details, see Figure 8.). The presence of PUFA in these two parasites can be explained by their salvage from the host. The only known apicomplexans completely lacking the apicoplast are eugregarines (like *Gregarina niphandrodes*) [84] and members of the genus *Cryptosporidium* [85,86]. Due to the loss of the complete plastidial FAS II pathway and all desaturases and elongases, *C. parvum* displays the most reduced enzymatic equipment for FA synthesis and modification among apicomplexans. Like *C. velia*, *T. gondii* and *E. tenella* have retained the majority of the enzymatic equipment, except for Δ5/6 and Δ12/15 desaturases. While *T. gondii* uses three elongases and only a single Δ9 desaturase [54], *C. velia* and *V. brassicaformis* encode six and eight elongases, respectively, and two Δ9 desaturases. The loss of photosynthesis and switch to parasitism have been accompanied by losses of enzymes involved in FA synthesis in a lineage-specific pattern. The ancestral state was most likely equipped as the typical photosynthetic primary producer.

Cytosolic S/MUFA elongases branch along with stramenopiles (Figure 4), suggesting the possible transfer from a hypothetical tertiary stramenopile endosymbiont linked to the origin of the chromerid plastid [2,4,87,88]. The group of S/MUFA elongases branching along with apicomplexans reflects the common origin of the chromerids and apicomplexans in the frame of the myzozoan lineage. A single gene of *C. velia* (Cvel_6334.t1) constitutes a long branch, sister to haptophytes. Similarly, a single PUFA elongase found in *C. velia* (Cvel_1604.t1) is related to stramenopiles, in contrast to three genes from *V. brassicaformis*, with apicomplexan relatives. Long branching and orphan phylogenetic positions are common phenomena for genes of *C. velia*. It appears that the alga retained genes of ancient origin, acting as a collector of genetic fossils.

## 5. Conclusions

Sequence identification of particular enzymes involved in FA synthesis and their predicted localization (Table 1, Table 2, Table 3, Table 4 and Table 5) together with GC analysis of particular FAs (Figure 5) provided enough information for proposing a system of fatty acid biosynthesis, elongation, and desaturation in chromerids (Figure S4). The primary sources of energy and carbons for the synthesis of fatty acids in chromerids are photosynthesis and the Calvin cycle, as described in plants [56,79]. We propose that the FAS II system localized in the plastid produces de novo short saturated fatty acids—mainly C16:0 (palmitic acid), followed by C18:0 (stearic acid) and C14:0 (myristic acid)—and exports them to the cytosol as fatty acids or in association with ACP. Fatty acids with ACP are then modified by the set of desaturases and elongases. Most enzymes participating in the final steps of fatty acid synthesis are either localized in the cytosol or associated with the membrane of the endoplasmic reticulum, with the exclusion of one ∆9 desaturase (Cvel21149), one ω-desaturase (Cvel22707), which are putatively localized in the plastid (Table 1, Table 2, Table 3, Table 4 and Table 5), as described in plants [24], and one front-end desaturase (Vbra 20473) probably localized in the mitochondrion.

## Figures and Tables

**Table 1:** Ketoacyl synthases (KSs), elongases, and desaturases in chromerids and their predicted subcellular location. ER—endoplasmic reticulum.

Ketoacyl Synthases	Accession	Length (AA)	SignalP 3.0	TargetP 1.1	TargetP (trimmed SP)	ASAFind	Pred. Localization
Type I FAS	Cvel_12437.t1	11,656	-	-	-	Cytosol/ER	Cytosol
	Cvel_19857.t1	6729	-	-	-	Cytosol/ER	Cytosol
	Vbra_3754.t1	7413	-	-	-	Cytosol/ER	Cytosol
	Vbra_4545.t1	9001	-	-	-	Cytosol/ER	Cytosol
	Vbra_18624.t1	8182	-	-	-	Cytosol/ER	Cytosol
Type II FAS	Cvel_3910.t1	463	SP (1.000, L19)	M (0.579, R5, L38)	M (0.625, R5, L19)	Plastid/mitoch	Plastid
	Vbra_22540.t1	504	SP (0.920, L33)	SP (0.968, R1, L33)	M (0.653, R4, L19)	Plastid	Plastid
ACR-B coupled	Cvel_8275.t1	4523	Anch (0.716, L26)	M (0.778, R3, L26)	-	Cytosol/ER	ER/ Microsome memb.
	Cvel_22311.t1	4384	Anch (0.868, L46)	M (0.706, R4, L15)	-	Cytosol/ER	ER/ Microsome memb.
Other KSs	Cvel_11706.t1	2033	-	M (0.489, R5, L21)		Cytosol/ER	Cytosol
	Cvel_12573.t1	2656	-	-		Cytosol/ER	Cytosol
	Cvel_15255.t1	2854	SP (0.877, L29)	M (0.333, R5, L17)	M (0.669, R3, L7)	Cytosol/ER	Plastid
	Cvel_17953.t1	1403	-	-	-	Cytosol/ER	Cytosol
	Cvel_18613.t1	1930	-	-	-	Cytosol/ER	Cytosol
	Cvel_19321.t1	3353	-	-	-	Cytosol/ER	Cytosol
	Cvel_19611.t1	6404	-	-	-	Cytosol/ER	Cytosol
	Cvel_19874.t1	528	-	-	-	Cytosol/ER	Cytosol
	Cvel_22375.t1	2562	-	-	-	Cytosol/ER	Cytosol
	Cvel_23369.t1	2489	-	M (0.494, R5, L50)	-	Mitochondrion	Mitochondrion
	Cvel_24220.t1	1093	-	-	-	Cytosol/ER	Cytosol
	Cvel_24323.t1	782	-	-	-	Cytosol/ER	Cytosol
	Cvel_26639.t1	2832	-	M (0.683, R4, L11)	-	Cytosol/ER	Cytosol/Mitochondrion
	Cvel_28742.t1	2860	-	SP (0.546, R5, L17)	-	Cytosol/ER	Cytosol
	Cvel_32860.t1	565	-	-	-	Cytosol/ER	Cytosol
	Cvel_33368.t1	1048	-	SP (0.447, R5, L24)	-	Cytosol/ER	Cytosol

**Table 2:** Ketoacyl synthases, elongases, and desaturases in chromerids and their predicted subcellular location.

Ketoacyl Synthases	Accession	Length (AA)	SignalP 3.0	TargetP 1.1	TargetP (trimmed SP)	ASAFind	Pred. Localization
Other KSs	Cvel_33369.t1	720	-	-	-	Cytosol/ER	Cytosol
	Cvel_33747.t1	1598	-	-	-	Cytosol/ER	Cytosol
	Cvel_3424.t1	4498	-	-	-	Cytosol/ER	Cytosol
	Cvel_34436.t1	1211	-	-	-	Cytosol/ER	Cytosol
	Cvel_34654.t1	1165	-	-	-	Cytosol/ER	Cytosol
	Cvel_34826.t1	1174	-	-	-	Cytosol/ER	Cytosol
	Cvel_35138.t1	1013	-	-	-	Cytosol/ER	Cytosol
	Cvel_35394.t1	1007	-	-	-	Cytosol/ER	Cytosol
	Cvel_35394.t2	968	-	-	-	Cytosol/ER	Cytosol
	Cvel_36416.t1	675	-	-	-	Cytosol/ER	Cytosol
	Cvel_36618.t1	349	-	-	-	Cytosol/ER	Cytosol
	Cvel_3734.t1	4498	-	-	-	Cytosol/ER	Cytosol
	Cvel_6161.t1	6550	-	-	-	Cytosol/ER	Cytosol
	Cvel_6212.t1	2635	-	-	-	Cytosol/ER	Cytosol
	Cvel_678.t1	2469	-	-	-	Cytosol/ER	Cytosol
	Vbra_1266.t1	6691	-	-	-	Cytosol/ER	Cytosol
	Vbra_1193.t1	1857	-	-	-	Cytosol/ER	Cytosol
	Vbra_12045.t1	3268	-	-	-	Cytosol/ER	Cytosol
	Vbra_12047.t1	1204	-	SP (0.777, R4, L19)	-	Cytosol/ER	Cytosol
	Vbra_135.t1	1603	-	-	-	Cytosol/ER	Cytosol
	Vbra_14267.t1	1337	-	-	-	Cytosol/ER	Cytosol
	Vbra_1496.t1	3575	-	-	-	Cytosol/ER	Cytosol
	Vbra_16257.t1	1220	-	-	-	Cytosol/ER	Cytosol
	Vbra_21227.t1	6651	-	-	-	Cytosol/ER	Cytosol
	Vbra_2242.t1	995	SP (0.959, L16)	M (0.442, R5, L12)	-	Cytosol/ER	ER/Microsome lumen

**Table 3:** Ketoacyl synthases, elongases, and desaturases in chromerids and their predicted subcellular location.

Ketoacyl Synthases	Accession	Length (AA)	SignalP 3.0	TargetP 1.1	TargetP (trimmed SP)	ASAFind	Pred. Localization
Other KSs	Vbra_22449.t1	1668	-	-	-	Cytosol/ER	Cytosol
	Vbra_23040.t1	4167	-	-	-	Cytosol/ER	Cytosol
	Vbra_23145.t1	480	-	-	-	Cytosol/ER	Cytosol
	Vbra_23180.t1	2233	-	-	-	Cytosol/ER	Cytosol
	Vbra_23216.t1	301	-	-	-	Cytosol/ER	Cytosol
	Vbra_23250.t1	1732	-	-	-	Cytosol/ER	Cytosol
	Vbra_23378.t1	1092	-	-	-	Cytosol/ER	Cytosol
	Vbra_424.t1	1469	-	-	-	Cytosol/ER	Cytosol
	Vbra_425.t1	1098	-	-	-	Cytosol/ER	Cytosol
	Vbra_428.t1	3041	-	-	-	Cytosol/ER	Cytosol
	Vbra_460.t1	3015	-	-	-	Cytosol/ER	Cytosol
	Vbra_483.t1	2820	-	SP (0.479, R4, L43)	-	Cytosol/ER	Cytosol
	Vbra_50.t1	526	-	-	-	Cytosol/ER	Cytosol
	Vbra_502.t1	710	SP (0.871, L20)	SP (0.479, R4, L20)	-	Plastid	Plastid/ER
	Vbra_51.t1	3829	-	-	-	Cytosol/ER	Cytosol
	Vbra_6248.t1	1297	-	-	-	Cytosol/ER	Cytosol
	Vbra_7032.t1	2189	-	-	-	Cytosol/ER	Cytosol
	Vbra_7414.t1	461	-	-	-	Cytosol/ER	Cytosol
	Vbra_8741.t1	1101	-	-	-	Cytosol/ER	Cytosol
**FASII (remaining) parts)**	**Accession**	**Length (AA)**	**SignalP 3.0**	**TargetP 1.1**	**TargetP (trimmed SP)**	**ASAFind**	**Pred. Localization**
Acyl transferase	Cvel_4616.t1	349	SP (0.983, L20)	SP (0.729, L19, R3)	M 0.526 (R5, L19)	Plastid	Plastid
	Vbra_21812.t1	362	SP (0.996, L21)	SP (0.694, L20, R3)	M 0.635 (R4, L15)	Plastid	Plastid
Enoyl reductase	Cvel_5563.t1	355	SP (0.958, L20)	SP (0.785, L19, R3)	M 0.912 (R2, L23)	Plastid	Plastid
	Vbra_11747.t1	412	SP (0.996, L22)	SP (0.982, L33, R1)	M 0.919 (R1, L20)	Cytosol/ER	Plastid
Ketoacyl reductase (KR)	Cvel_3619.t1	392	SP (0.912, L19)	SP (0.915, L18, R1)	M 0.700 (R3, L17)	Plastid	Plastid
	Vbra_710.t1	338	SP (0.996, L22)	SP (0.943, L21, R1)	M 0.766 (R3, L10)	Plastid	Plastid
Dehydrogenase	Cvel_14912.t1	213	SP (0.849, L18)	SP (0.884, L17, R2)	M 0.526 (R5, L19)	Plastid	Plastid
	Vbra_19455.t1	225	SP (0.993, L25)	SP (0.800, L22, R2)	M 0.827 (R2, L43)	Plastid	Plastid

**Table 4:** Ketoacyl synthases, elongases, and desaturases in chromerids and their predicted subcellular location. S/MUFAs, saturated and monounsaturated FAs, PUFA, poly-unsaturated FAs.

Elongases							
S/MUFA	Cvel_6334.t1	193	SP (0.960, L16)	SP (0.861, R2, L16)	M (0.689, R3, L5)	Cytosol/ER	Plastid
	Cvel_13090.t1	525	Anch (0.838, L41)	-	-	Cytosol/ER	ER/Microsome memb.
	Cvel_14249.t1	884	Anch (0.791, L39)	-	-	Cytosol/ER	ER/Microsome memb.
	Cvel_15641.t1	2131	Anch (0.606, L50)	-	-	Cytosol/ER	ER/Microsome memb.
	Cvel_12461.t1	415	-	-	-	Cytosol/ER	Cytosol
	Vbra_3929.t1	307	-	-	-	Cytosol/ER	Cytosol
	Vbra_9666.t1	885	SP (0.675, L24)	SP (0.622, R3, L40)	S (0.950, R1, L16)	Cytosol/ER	ER/Microsome lumen
	Vbra_11085.t1	281	Anch (0.560, L16)	-	-	Cytosol/ER	ER/Microsome memb.
	Vbra_12050.t1	330	Anch (0.600, L41)	-	-	Cytosol/ER	ER/Microsome memb.
	Vbra_13163.t1	363	-	-	-	Cytosol/ER	Cytosol
PUFA	Cvel_1604.t1	297	-	-	-	Cytosol/ER	Cytosol
	Vbra_3441.t1	363	Anch (0.984, L55)	-	-	Cytosol/ER	ER/Microsome memb.
	Vbra_11843.t1	306	Anch (0.986, L63)	-	-	Cytosol/ER	ER/Microsome memb.
	Vbra_16961.t1	291	Anch (0.638, L62)	-	-	Cytosol/ER	ER/Microsome memb.

**Table 5:** Ketoacyl synthases, elongases, and desaturases in chromerids and their predicted subcellular location.

Desaturases	Accession	Length (AA)	SignalP 3.0	TargetP 1.1	TargetP (trimmed SP)	ASAFind	Pred. Localization
Omega	Cvel_2615.t1	461	-	-	-	Cytosol/ER	Cytosol
	Cvel_21003.t1	440	-	-	-	Cytosol/ER	Cytosol
	Cvel_22707.t1	425	SP (0.999, L17)	M/SP (0.631/0.771,L17, R5)	M (0.852, R2, L71)	Plastid	Plastid
	Vbra_7407.t1	445	-	M (0.564, L12, R5)	-	Cytosol/ER	Cytosol
	Vbra_15192.t1	465	-	-	-	Cytosol/ER	Cytosol
	Vbra_20615.t1	447	-	-	-	Cytosol/ER	Cytosol
Delta 9	Cvel_14249.t1	884	Anch (0.791, L39)	-	-	Cytosol/ER	ER/Microsome memb.
	Cvel_21149.t1	395	SP (0.910, L37)	SP (0.979, L37, R1)	M 0.814 (R2, L6)	Cytosol/ER	Plastid
	Vbra_9666.t1	885	SP (0.675, L40)	SP (0.622, L40, R3)	-	Cytosol/ER	ER/Microsome lumen
	Vbra_15445.t1	565	-	M (0.549, L19, R5)	-	Cytosol/ER	Cytosol
Front-end	Cvel_8966.t1	507	-	-	-	Cytosol/ER	Cytosol
	Cvel_17413.t1	543	-	-	-	Cytosol/ER	Cytosol
	Vbra_17659.t1	439	-	-	-	Cytosol/ER	Cytosol
	Vbra_20473.t1	437	-	M (0.738, L17, R3)	-	Mitochondrion	Mitochondrion
**Acetyl-CoA Carboxylases**	**Accession**	**Length (AA)**	**SignalP 3.0**	**TargetP 1.1**	**TargetP (trimmed SP)**	**ASAFind**	**Pred. Localization**
	Cvel_530.t2	2097	-	M (0.478, L18, R5)	-	Cytosol/ER	Cytosol
	Cvel_25292.t1	1651	-	-	-	Cytosol/ER	Cytosol
	Vbra_9562.t1	2702	-	-	-	Cytosol/ER	Cytosol
	Vbra_15163.t1	2146	SP (0.999, L19)	SP (0.828, L19, R2)	M 0.833 (R2, L20)	Cytosol/ER	Plastid

## Supplementary Materials

The following are available online at https://www.mdpi.com/2218-273X/10/8/1102/s1: Figure S1. The Δ-9 desaturases Likelihood/Bayesian tree. Figure S2. The Δ5/6 (front-end) desaturase Likelihood/Bayesian tree. Figure S3. The omega (Δ12/15) desaturase Likelihood/Bayesian tree. Figure S4. The model of fatty acid biosynthesis in *Chromera velia* (A) and in *Vitrella brassicaformis* (B). Figure S5. Growing curves of *Chromera velia* treated by the various concentration of triclosan recorded by the Tecan instrument (*n* = 5). Figure S6. Spectra of fatty acid composition changes after triclosan treatment obtained by GC/FID. Figure S7 Transmission electron microscopy of *C. velia* grown in standard f/2 medium (A) and grown in f/2 medium with 333 µM triclosan (B), where the cell is deformed and the inner structure is collapsed. LD—lipid droplets; N—nucleus; P—plastid. Figure S8. The ratio of fatty acids shorter than C18 and C18 (blue) and longer than C18 (orange) in *C.velia* during nitrogen deprivation and repletion (*n* = 5). Figure S9. Maximum-likelihood phylogenetic tree of acetyl-CoA decarboxylases. Figure S10. The alignment of ACCases of chromerids, showing the first 408 amino acid residues, with the N-terminal motif similarity shared between plastid-targeted gene variants.
