# Supplementary material for: Fatty Acid Biosynthesis in Chromerids
Source: Biomolecules. 2020 Jul 24;10(8):1102. doi: 10.3390/biom10081102 (PMC7464705; doi:10.3390/biom10081102)
Supplement: Supplementary file 1 [file biomolecules-10-01102-s001.zip › Supplementary Materials captions.docx]

**Supplementary Materials:** The following are available online at [www.mdpi.com/xxx/s1](http://www.mdpi.com/xxx/s1)

**Figure S1.** The Δ-9 desaturases Likelihood/ Bayesian tree. Supported branches are labeled with corresponding values of bootstrap and posterior probability. Predicted subcellular locations are displayed.

**Figure S2.** The Δ5/6 (front-end) desaturase Likelihood/ Bayesian tree. Supported branches are labeled with corresponding values of bootstrap and posterior probability. Predicted subcellular locations are displayed.

**Figure S3.** The omega (Δ12/15) desaturase Likelihood/ Bayesian tree. Supported branches are labeled with corresponding values of bootstrap and posterior probability. Predicted subcellular locations are displayed.

**Figure S4.** The model of fatty acid biosynthesis in *Chromera velia* (A) and in *Vitrella brassicaformis* (B).

**Figure S5.** Growing curves of *Chromera velia* treated by the various concentration of Triclosan recorded by the Tecan instrument (n=5).

**Figure S6.** Spectra of fatty acid composition changes after Triclosan treatment obtained by GC FID

**Figure S7** Transmission electron microscopy of *C. velia* grew in standard f/2 medium (A) and grown in f/2 medium with 333 µM Triclosan (B), where the cell is deformed and the inner structure is collapsed. LD – lipid droplets; N – nucleus; P – plastid

**Figure S8.** The ratio of fatty acids shorter than C18 and C18 (blue) and longer than C18 (orange) in *C.velia* during nitrogen deprivation and repletion (n=5). Data were obtained by GC FID.

**Figure S9.** Maximum-likelihood phylogenetic tree of Acetyl-CoA decarboxylases. Supported branches are labeled with corresponding values of ML bootstraps and Bayesian posterior probabilities L.

**Figure S10.** The alignment of ACCases of chromerids showing the first 408 amino acid residues with the N-terminal motif similarity shared between plastid-targeted gene variants. Plastid targeted variants are highlighted in green, cytosolic in blue. The bright blue field highlights the actual functional-domain part of the genes.
