# Supplementary figures and images for: Fatty Acid Biosynthesis in Chromerids
Source: Biomolecules. 2020 Jul 24;10(8):1102. doi: 10.3390/biom10081102 (PMC7464705; doi:10.3390/biom10081102)

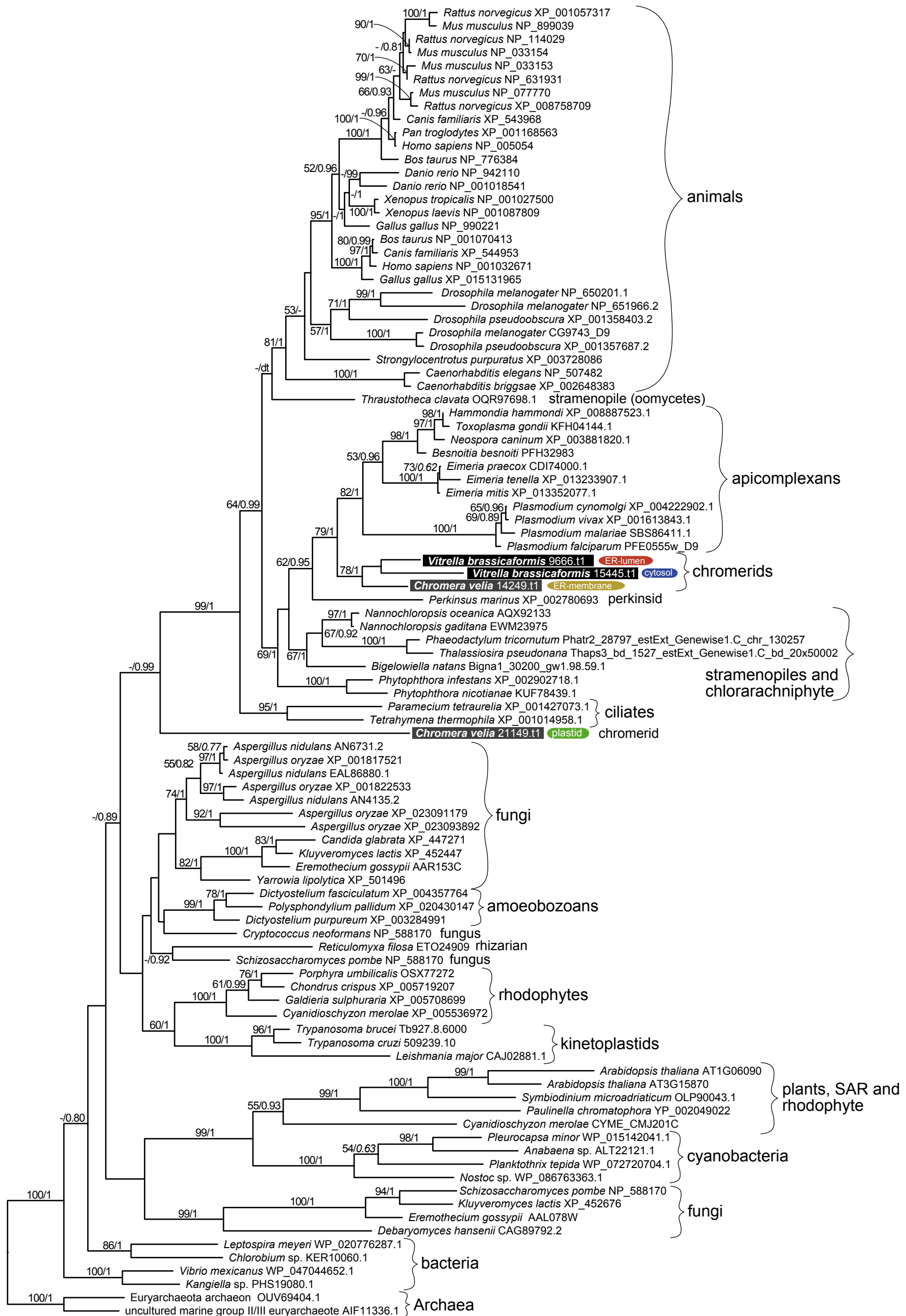

Supplement: Supplementary file 1 [file biomolecules-10-01102-s001.zip › FS1_D9_vobFIN1_Ktarg1_fixed1.pdf]

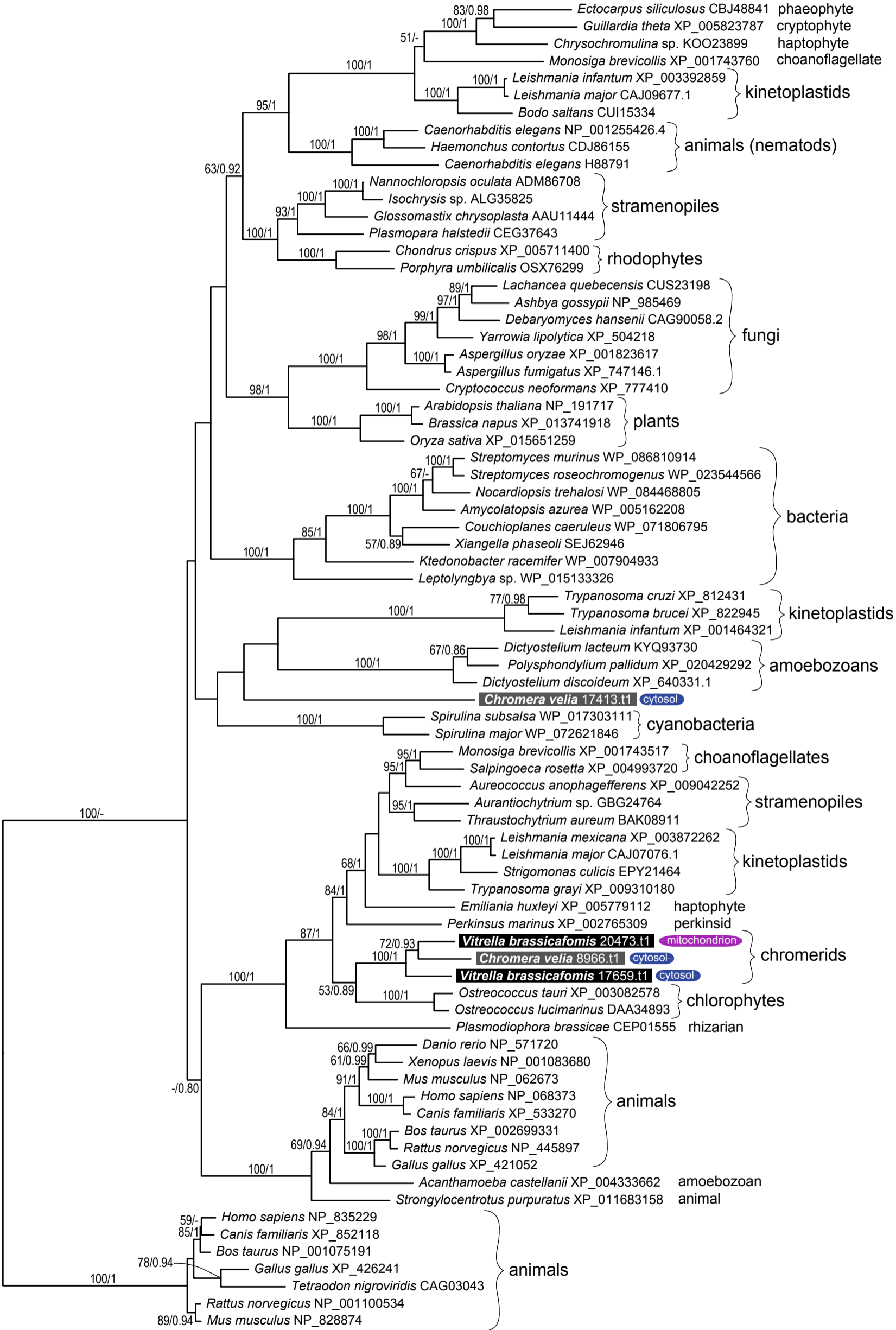

0.5

Supplement: Supplementary file 1 [file biomolecules-10-01102-s001.zip › FS2_front4_2_Kajtarg1.pdf]

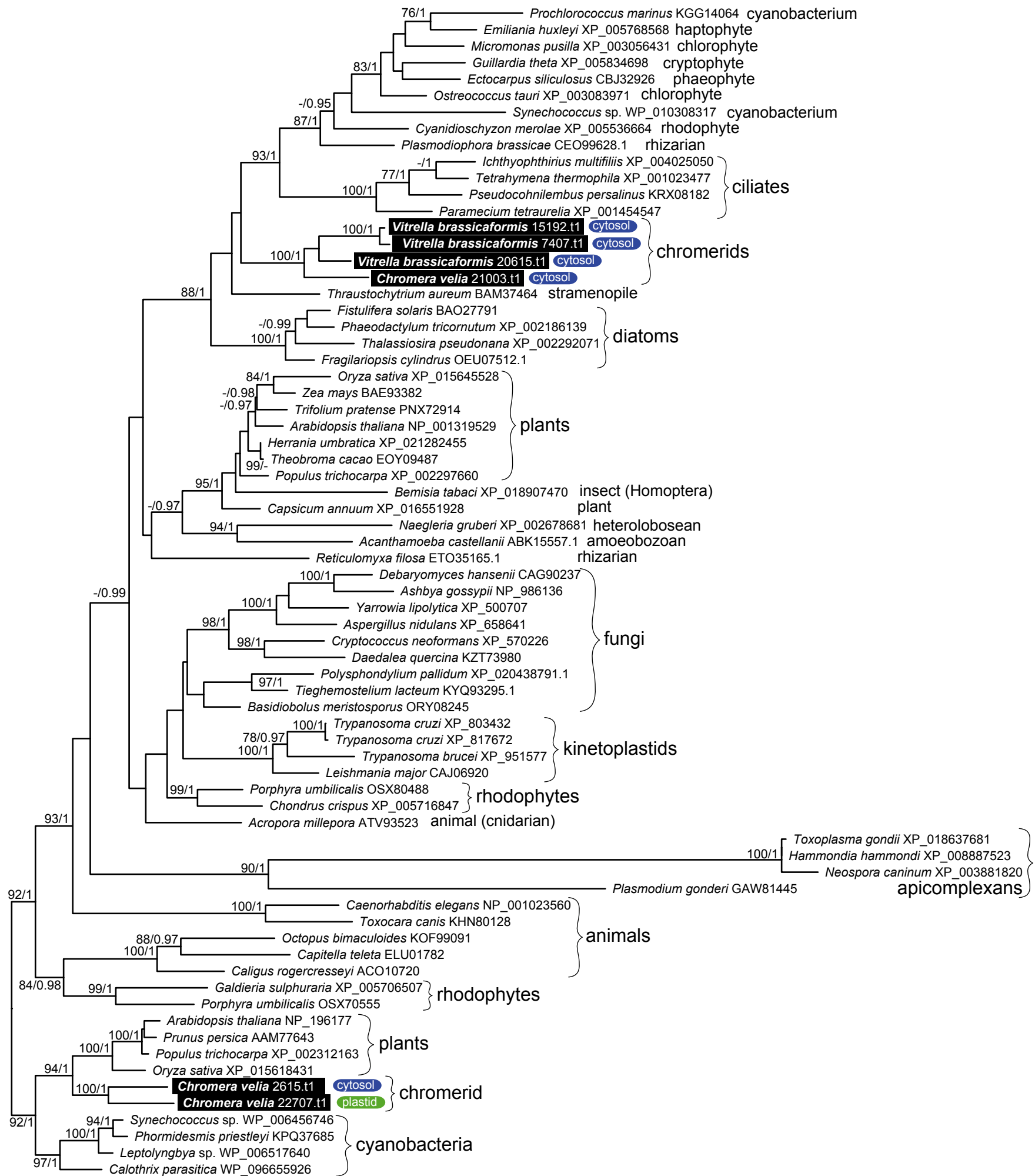

0.6

Supplement: Supplementary file 1 [file biomolecules-10-01102-s001.zip › FS3_omega_9_2finVOB_K1targ_BayML.pdf]

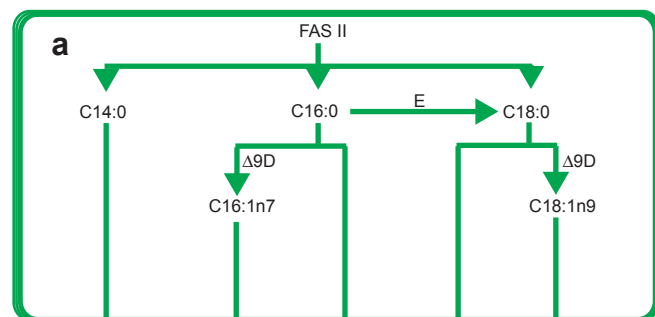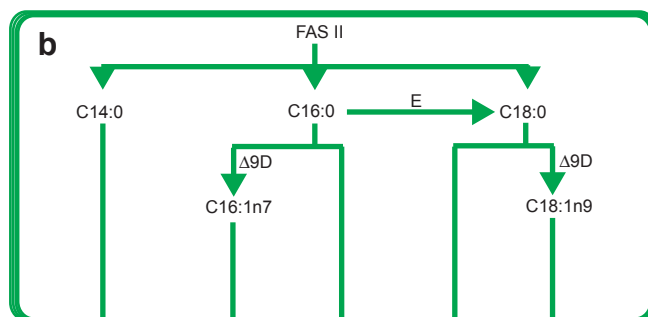

Supplement: Supplementary file 1 [file biomolecules-10-01102-s001.zip › FS4ab_ModelFABiosynthesis.pdf]

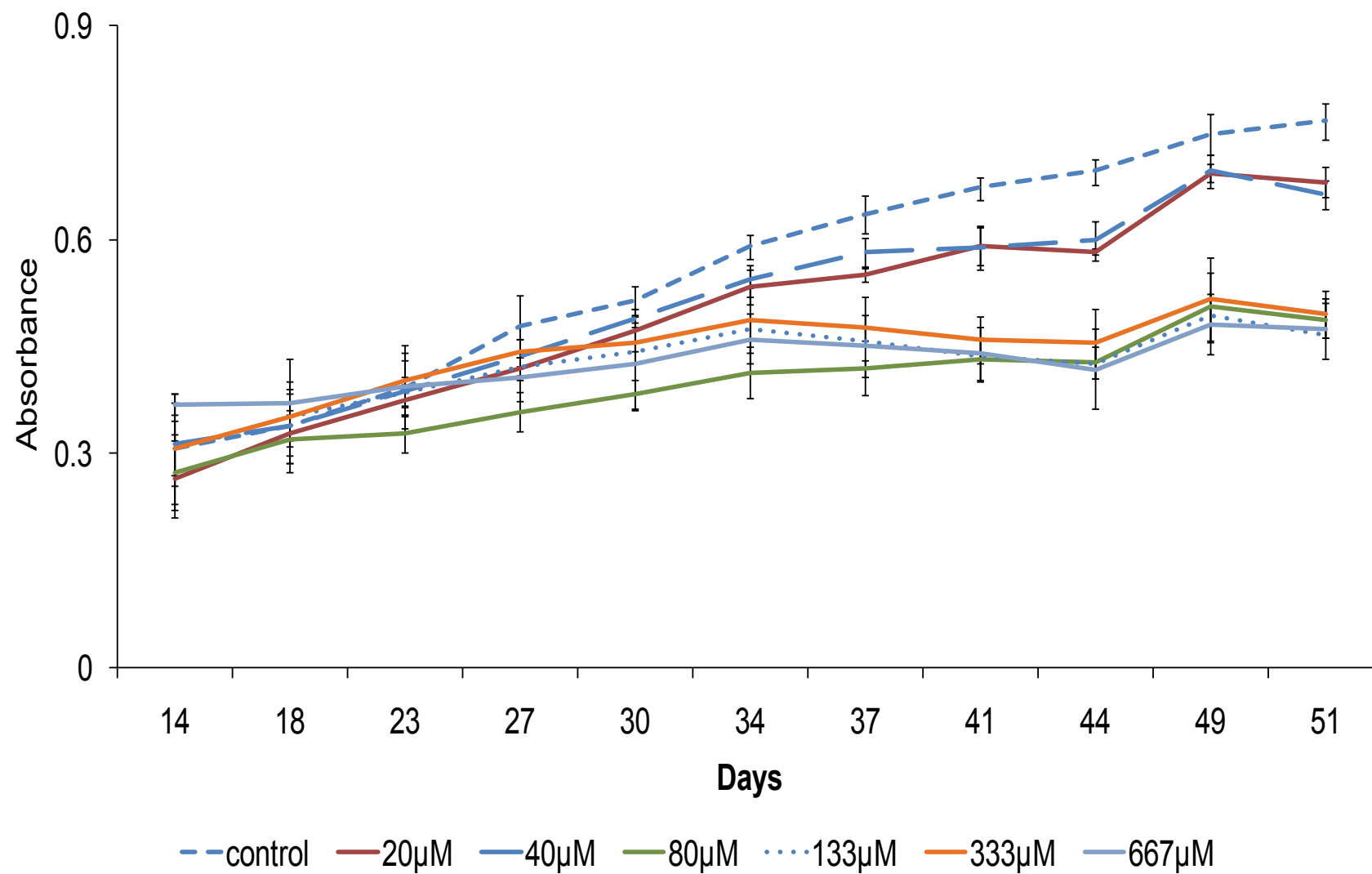

Supplement: Supplementary file 1 [file biomolecules-10-01102-s001.zip › FS5_TLC_growingcurves.pdf]

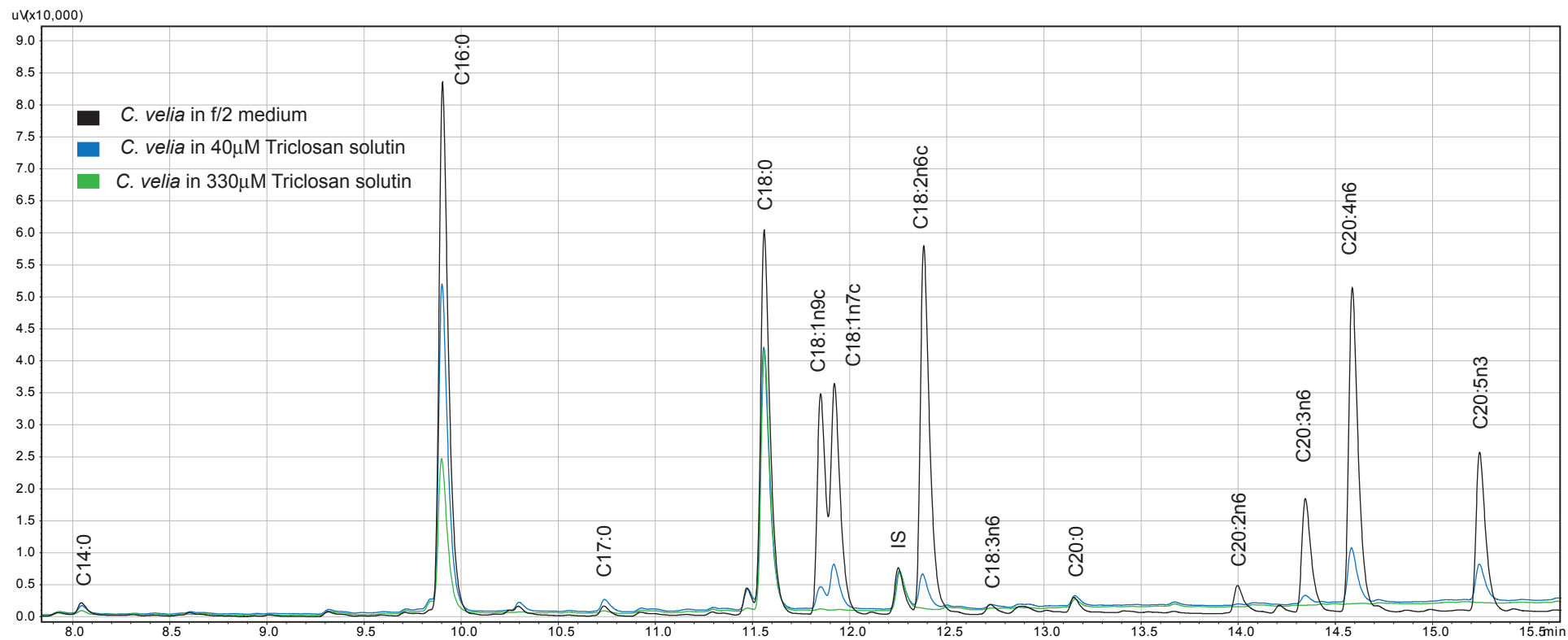

Supplement: Supplementary file 1 [file biomolecules-10-01102-s001.zip › FS6_TCL_FID.pdf]

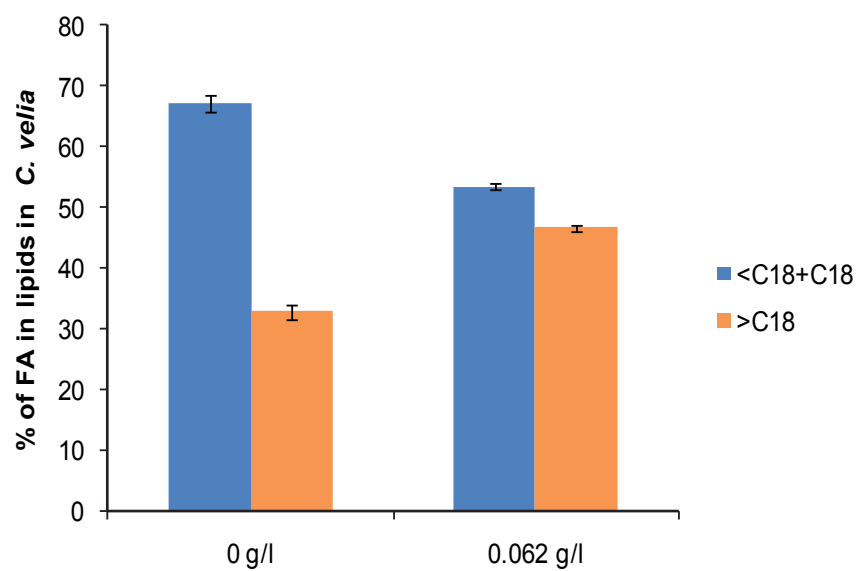

Supplement: Supplementary file 1 [file biomolecules-10-01102-s001.zip › FS8_Nitroge_FA.pdf]

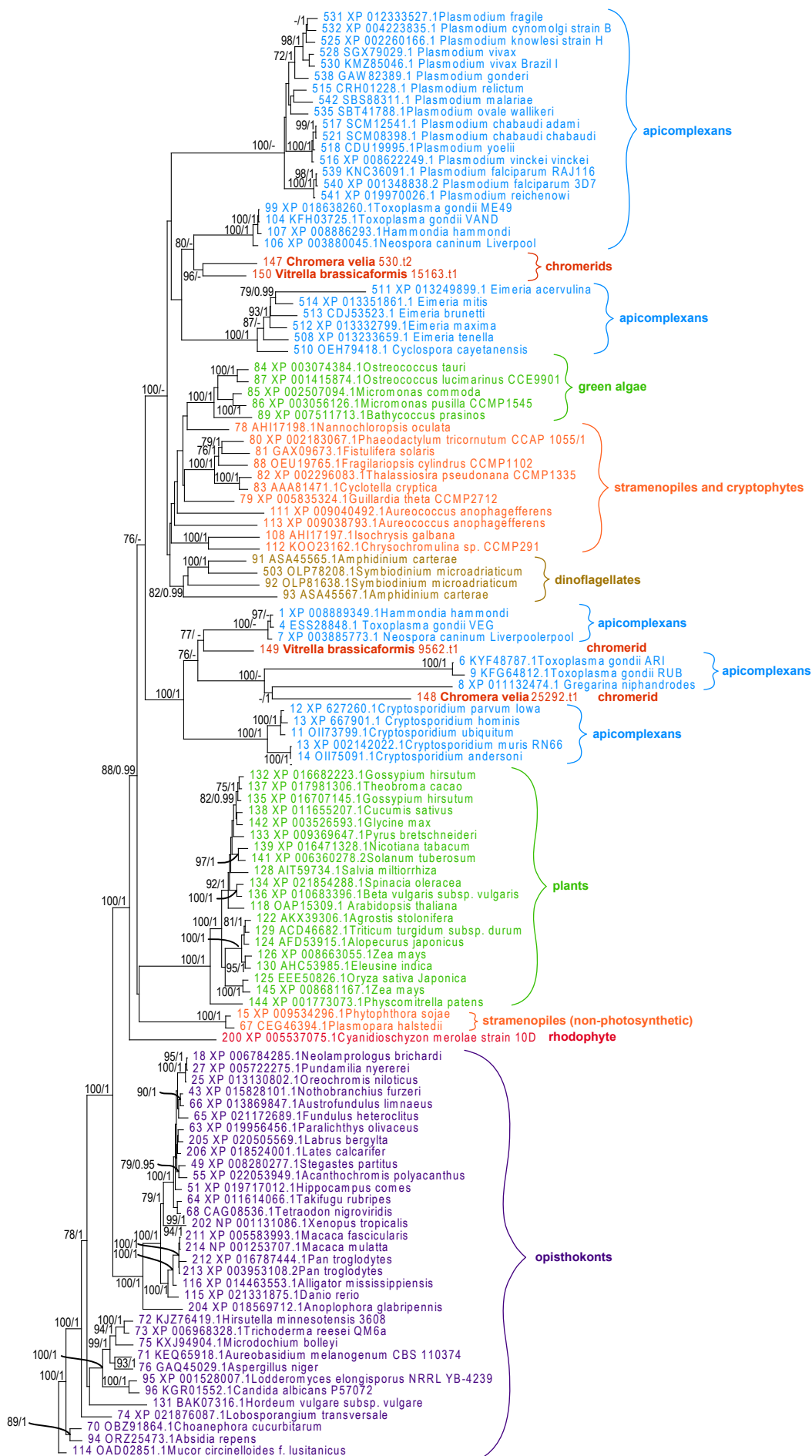

Supplement: Supplementary file 1 [file biomolecules-10-01102-s001.zip › FS9_ACCall31_8_17ed4alSEL2treeBAR_ML_Bay_1.pdf]

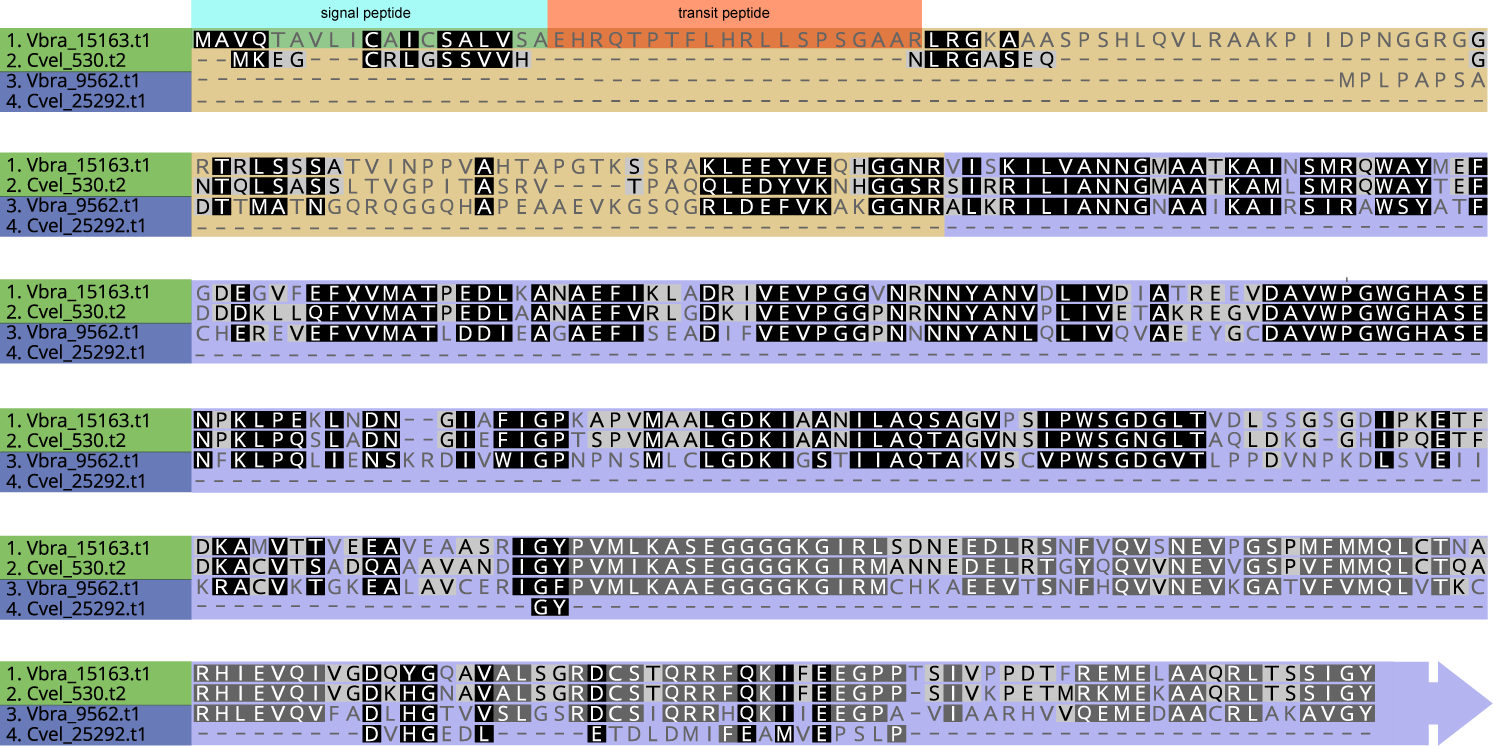

Supplement: Supplementary file 1 [file biomolecules-10-01102-s001.zip › FS10.png]

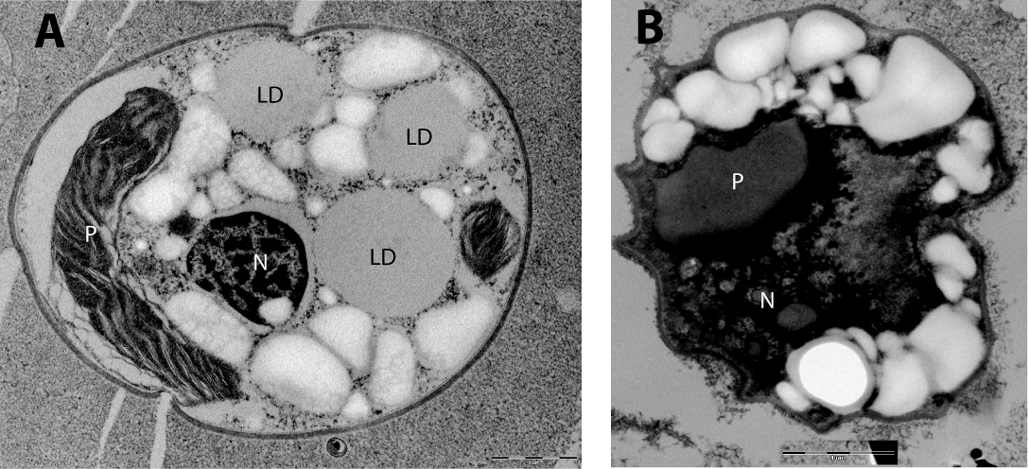

Supplement: Supplementary file 1 [file biomolecules-10-01102-s001.zip › FS7 AB_TEM_triclosan.tif]
